# 

**Published:** 1876-10

**Authors:** 


Vol. XXXIII.
Number 10.

October, 1876.

NEW SERIES
Vol. I.
Number 10.

THE

### CHICAGO MEDICAL Journal and Examiner

PUBLISHED MONTHLY.

**EDITOR:**
WILLIAM H. BYFORD, A.M., M.D.

**ASSOCIATE EDITORS:**

JAMES H. ETHERIDGE, M.D.

NORMAN BRIDGE, M.D.

JAS. NEVINS HYDE, A.M., M.D.

FERD. C. HOTZ, M.D.

Published under the auspices of the

*CHICAGO MEDICAL PRESS ASSOCIATION.*

**TERMS—FOUR DOLLARS PER ANNUM, IN ADVANCE.
POSTAGE FREE.**

FOLIA SUA AD SANITATEM GENTIUM

CHICAGO:
W. B. KEEN, COOKE & CO., Publishers,
Nos. 113 and 115 State Street.

HAZLITT & REED, PRINTERS, 172 & 174 CLARK ST., CHICAGO.

